# 2,2′-[(3-Bromo-4-hy­droxy-5-meth­oxy­phen­yl)methyl­idene]bis­(3-hy­droxy-5,5-dimethyl­cyclo­hex-2-en-1-one)

**DOI:** 10.1107/S1600536812037853

**Published:** 2012-09-08

**Authors:** V. Sughanya, N. Sureshbabu

**Affiliations:** a Department of Chemistry, Annamalai University, Annamalai Nagar 608 002, Tamil Nadu, India

## Abstract

In the title compound, C_24_H_29_BrO_6_, the dihedral angle between the cyclo­hexenone mean planes is 57.63 (2)° while the dihedral angles between the benzene ring and the cyclo­hexenone mean planes are 58.42 (2) and 69.08 (3)°. The two cyclo­hexenone rings both show an envelope conformation, with the C atom bearing two methyl groups as the flap atom in each ring. Two intra­molecular O—H⋯O hydrogen bonds occur. In the crystal, molecules are linked *via* pairs of O—H⋯O hydrogen bonds, forming inversion dimers.

## Related literature

For the synthesis of bis­dimedones, see: Vanag & Stankevich (1960 ▶); Hilderbrand & Weissleder (2007 ▶). For their pharmaceutical properties, see: Lambert *et al.* (1997 ▶); Poupelin *et al.* (1978 ▶); Hideo (1981 ▶); Selvanayagam *et al.* (1996 ▶); Jonathan *et al.* (1988 ▶). For crystal structures of related xanthene derivatives, see: Odabaşoğlu *et al.* (2008 ▶); Mehdi *et al.* (2011 ▶); Ravikumar *et al.* (2012 ▶); Sureshbabu & Sughanya (2012 ▶). For the assignment of ring conformations, see: Cremer & Pople (1975 ▶).
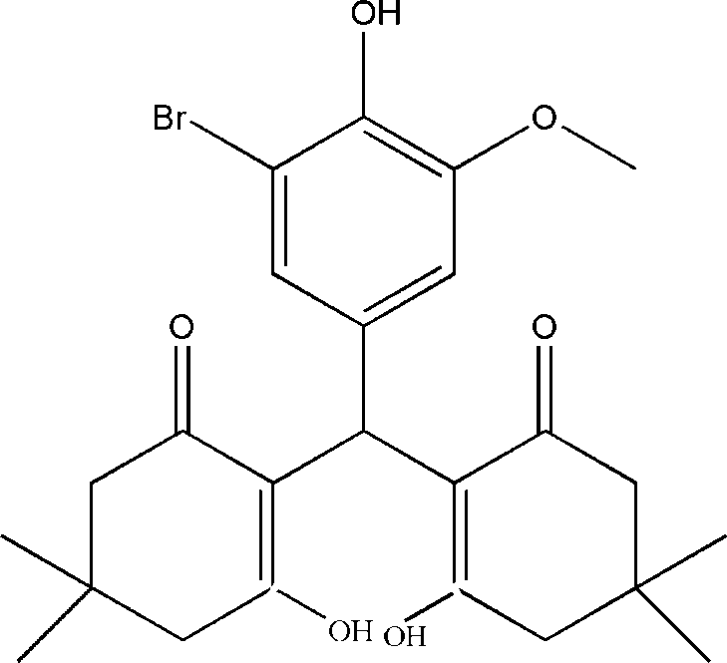


## Experimental

### 

#### Crystal data

C_24_H_29_BrO_6_
*M* *_r_* = 493.38
Monoclinic, 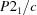

*a* = 11.7479 (4) Å
*b* = 19.3706 (6) Å
*c* = 11.5958 (4) Å
β = 118.365 (1)°
*V* = 2321.97 (13) Å^3^
*Z* = 4
Mo *K*α radiation
μ = 1.81 mm^−1^
*T* = 296 K
0.30 × 0.20 × 0.20 mm

#### Data collection

Bruker Kappa APEXII CCD diffractometer
Absorption correction: multi-scan (*SADABS*; Bruker, 2004 ▶) *T*_min_ = 0.604, *T*_max_ = 0.765
22187 measured reflections
4313 independent reflections
3395 reflections with *I* > 2σ(*I*)
*R*_int_ = 0.031

#### Refinement

*R*[*F* ^2^ > 2σ(*F* ^2^)] = 0.035
*wR*(*F* ^2^) = 0.095
*S* = 1.04
4313 reflections
285 parameters
H-atom parameters constrained
Δρ_max_ = 0.37 e Å^−3^
Δρ_min_ = −0.51 e Å^−3^

### 

Data collection: *APEX2* (Bruker, 2004 ▶); cell refinement: *APEX2* and *SAINT* (Bruker, 2004 ▶); data reduction: *SAINT* and *XPREP* (Bruker, 2004 ▶); program(s) used to solve structure: *SIR92* (Altomare *et al.*, 1993 ▶); program(s) used to refine structure: *SHELXL97* (Sheldrick, 2008 ▶); molecular graphics: *ORTEP-3* (Farrugia, 1997 ▶) and *Mercury* (Macrae *et al.*, 2008 ▶); software used to prepare material for publication: *SHELXL97*.

## Supplementary Material

Crystal structure: contains datablock(s) global, I. DOI: 10.1107/S1600536812037853/im2397sup1.cif

Structure factors: contains datablock(s) I. DOI: 10.1107/S1600536812037853/im2397Isup2.hkl

Supplementary material file. DOI: 10.1107/S1600536812037853/im2397Isup3.cml

Additional supplementary materials:  crystallographic information; 3D view; checkCIF report

## Figures and Tables

**Table 1:** Hydrogen-bond geometry (Å, °)

*D*—H⋯*A*	*D*—H	H⋯*A*	*D*⋯*A*	*D*—H⋯*A*
O5—H5*A*⋯O3^i^	0.82	2.12	2.852 (2)	149
O3—H3⋯O2	0.82	1.97	2.615 (2)	135
O1—H1⋯O4	0.82	1.82	2.640 (2)	174

Symmetry code: (i) 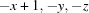.

**Fig. 1.**
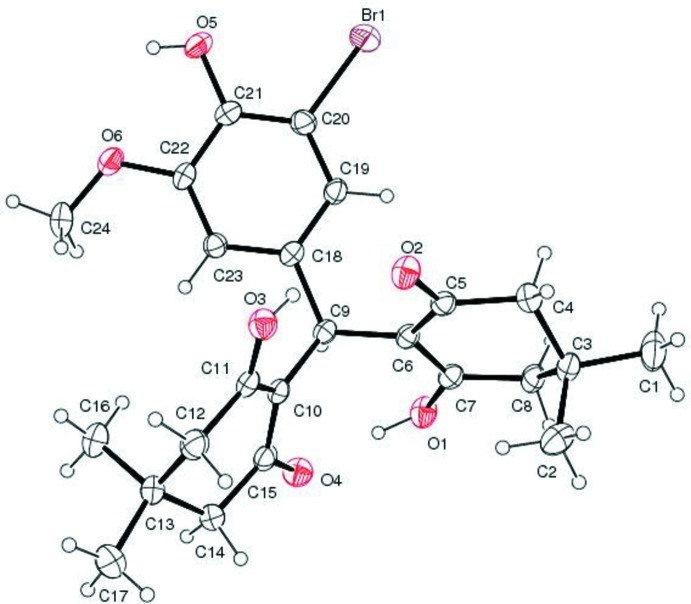
A view of the structure of the title compound. Displacement ellipsoids are drawn at the 50% probability level. Hydrogen atoms are shown as spheres of arbitrary radius.

**Fig. 2.**
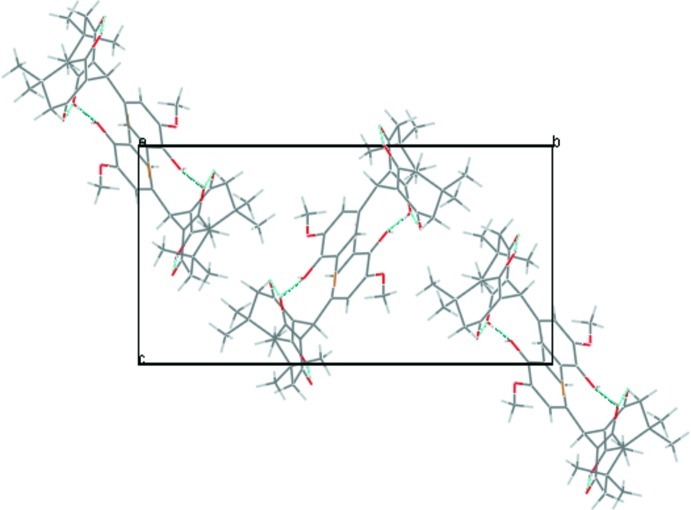
A view of the packing in the crystal structure, showing intermolecular as well as intramolecular O—H···O hydrogen bonds as dotted lines.

## supplementary crystallographic information

### Comment

Xanthene derivatives possess biological properties such as antibacterial, antiviral and anti-inflammatory activities (Jonathan *et al.*, 1988) and are used in medicine. Several methods have been reported in the literature for the synthesis of the title compound (Vanag & Stankevich, 1960; Hilderbrand & Weissleder, 2007). In view of the importance of the title compound, we herein report its crystal structure.

In the title compound, the cyclohexenone rings C3–C8 and C10–C15 both adopt envelope conformations, with flap atoms C3 and C13, respectively. The dihedral angle between the two cyclohexenone planes Q(C4/C5/C6/C7/C8) and R(C10/C11/C12/C14/C15) is 57.63 (2)°. The dihedral angle between the phenyl ring P(C18-C23) and the cyclohexenone planes Q and R are 58.42 (2)° and 69.08 (3)°, respectively. The hydroxy and carbonyl oxygen atoms face to each other and are oriented to allow for the formation of intermolecular as well as intramolecular O—H···O hydrogen bonds (Table 1, Fig.2), typical for xanthene derivatives.

### Experimental

The title compound was prepared in single stage. A mixture of 3-bromo-4-hydroxy-5-methoxy-benzaldehyde (1.84 g, 8 mmol), 5,5-dimethyl cyclohexane-1,3-dione (2.24 g, 16 mmol) and 20 ml of ethanol was heated to 70°C for about 10 minutes. The reaction mixture was allowed to cool to room temperature and the resulting title compound 2,2'-((3-Bromo-4-hydroxy -5-methoxyphenyl)methylene)bis(3-hydroxy-5,5-dimethylcyclohex-2-en-1-one) was filtered and dried. The crystal used for data collection was obtained by crystallisation from ethanol at room temperature.(m.pt. 491 K, Yield 85% ).

### Refinement

All hydrogen atoms were identified from difference electron density peaks and subsequently treated as riding atoms with d(Csp^2^—H) = 0.93 Å, d(Cmethyl—H) = 0.96 Å, d(Cmethylene—H) = 0.97 Å, d(Cmethine—H) = 0.98 Å; d(O—H) = 0.82 Å; U_iso_(H) = x U_eq_(C,O), where x = 1.5 for methyl H and 1.2 for all other H atoms.

### Crystal data

**Table:**

C_24_H_29_BrO_6_	*F*(000) = 1024
*M**_r_* = 493.38	*D*_x_ = 1.411 Mg m^−^^3^
Monoclinic, *P*2_1_/*c*	Melting point: 491 K
Hall symbol: -P 2ybc	Mo *K*α radiation, λ = 0.71073 Å
*a* = 11.7479 (4) Å	Cell parameters from 7579 reflections
*b* = 19.3706 (6) Å	θ = 2.1–25.4°
*c* = 11.5958 (4) Å	µ = 1.81 mm^−^^1^
β = 118.365 (1)°	*T* = 296 K
*V* = 2321.97 (13) Å^3^	Block, yellow
*Z* = 4	0.30 × 0.20 × 0.20 mm

### Data collection

**Table:**

Bruker Kappa APEXII CCD diffractometer	4313 independent reflections
Radiation source: fine-focus sealed tube	3395 reflections with *I* > 2σ(*I*)
Graphite monochromator	*R*_int_ = 0.031
ω and φ scan	θ_max_ = 25.5°, θ_min_ = 2.1°
Absorption correction: multi-scan (*SADABS*; Bruker, 2004)	*h* = −14→14
*T*_min_ = 0.604, *T*_max_ = 0.765	*k* = −23→19
22187 measured reflections	*l* = −14→13

### Refinement

**Table:**

Refinement on *F*^2^	Primary atom site location: structure-invariant direct methods
Least-squares matrix: full	Secondary atom site location: difference Fourier map
*R*[*F*^2^ > 2σ(*F*^2^)] = 0.035	Hydrogen site location: inferred from neighbouring sites
*wR*(*F*^2^) = 0.095	H-atom parameters constrained
*S* = 1.04	*w* = 1/[σ^2^(*F*_o_^2^) + (0.0471*P*)^2^ + 1.1375*P*] where *P* = (*F*_o_^2^ + 2*F*_c_^2^)/3
4313 reflections	(Δ/σ)_max_ = 0.002
285 parameters	Δρ_max_ = 0.37 e Å^−^^3^
0 restraints	Δρ_min_ = −0.51 e Å^−^^3^

### Special details

**Table:**

Geometry. All esds (except the esd in the dihedral angle between two l.s. planes) are estimated using the full covariance matrix. The cell esds are taken into account individually in the estimation of esds in distances, angles and torsion angles; correlations between esds in cell parameters are only used when they are defined by crystal symmetry. An approximate (isotropic) treatment of cell esds is used for estimating esds involving l.s. planes.
Refinement. Refinement of F^2^ against ALL reflections. The weighted R-factor wR and goodness of fit S are based on F^2^, conventional R-factors R are based on F, with F set to zero for negative F^2^. The threshold expression of F^2^ > 2sigma(F^2^) is used only for calculating R-factors(gt) etc. and is not relevant to the choice of reflections for refinement. R-factors based on F^2^ are statistically about twice as large as those based on F, and R- factors based on ALL data will be even larger.

### Fractional atomic coordinates and isotropic or equivalent isotropic displacement parameters (Å^2^)

**Table:**

	*x*	*y*	*z*	*U*_iso_*/*U*_eq_	
C1	1.2613 (3)	0.26315 (17)	0.2601 (3)	0.0659 (9)	
H1A	1.2246	0.3011	0.2005	0.099*	
H1B	1.2871	0.2277	0.2194	0.099*	
H1C	1.3354	0.2788	0.3383	0.099*	
C2	1.1247 (3)	0.28939 (14)	0.3665 (3)	0.0577 (7)	
H2A	1.0575	0.2722	0.3835	0.087*	
H2B	1.0948	0.3300	0.3128	0.087*	
H2C	1.1994	0.3007	0.4479	0.087*	
C3	1.1605 (2)	0.23420 (13)	0.2953 (2)	0.0417 (6)	
C4	1.0407 (2)	0.21187 (13)	0.1703 (2)	0.0397 (5)	
H4A	0.9988	0.2525	0.1187	0.048*	
H4B	1.0674	0.1831	0.1190	0.048*	
C5	0.9453 (2)	0.17286 (11)	0.1961 (2)	0.0328 (5)	
C6	0.98674 (19)	0.12637 (11)	0.3011 (2)	0.0306 (5)	
C7	1.1163 (2)	0.12824 (11)	0.3968 (2)	0.0346 (5)	
C8	1.2146 (2)	0.17062 (13)	0.3816 (2)	0.0407 (6)	
H8A	1.2567	0.1417	0.3448	0.049*	
H8B	1.2801	0.1853	0.4678	0.049*	
C9	0.89559 (19)	0.07694 (11)	0.3184 (2)	0.0303 (5)	
H9	0.9536	0.0432	0.3824	0.036*	
C10	0.8246 (2)	0.10924 (11)	0.3857 (2)	0.0310 (5)	
C11	0.7081 (2)	0.14463 (11)	0.3181 (2)	0.0341 (5)	
C12	0.6363 (2)	0.17136 (13)	0.3861 (3)	0.0460 (6)	
H12A	0.5443	0.1697	0.3257	0.055*	
H12B	0.6597	0.2193	0.4093	0.055*	
C13	0.6627 (2)	0.13151 (14)	0.5090 (3)	0.0468 (6)	
C14	0.8083 (2)	0.12723 (14)	0.5938 (2)	0.0449 (6)	
H14A	0.8404	0.1723	0.6321	0.054*	
H14B	0.8274	0.0952	0.6651	0.054*	
C15	0.8790 (2)	0.10448 (12)	0.5219 (2)	0.0365 (5)	
C16	0.6047 (3)	0.05929 (17)	0.4753 (3)	0.0685 (9)	
H16A	0.5151	0.0624	0.4109	0.103*	
H16B	0.6119	0.0375	0.5529	0.103*	
H16C	0.6504	0.0325	0.4410	0.103*	
C17	0.6055 (3)	0.16999 (19)	0.5846 (3)	0.0728 (9)	
H17A	0.6405	0.2158	0.6044	0.109*	
H17B	0.6268	0.1459	0.6647	0.109*	
H17C	0.5132	0.1724	0.5322	0.109*	
C18	0.8103 (2)	0.03309 (11)	0.1981 (2)	0.0310 (5)	
C19	0.8378 (2)	0.02503 (11)	0.0962 (2)	0.0353 (5)	
H19	0.9051	0.0498	0.0954	0.042*	
C20	0.7654 (2)	−0.01994 (12)	−0.0051 (2)	0.0379 (5)	
C21	0.6656 (2)	−0.05795 (12)	−0.0079 (2)	0.0348 (5)	
C22	0.6402 (2)	−0.05102 (12)	0.0971 (2)	0.0339 (5)	
C23	0.7111 (2)	−0.00615 (12)	0.1986 (2)	0.0329 (5)	
H23	0.6926	−0.0021	0.2678	0.039*	
C24	0.5090 (2)	−0.09167 (15)	0.1878 (3)	0.0520 (7)	
H24A	0.5833	−0.1052	0.2678	0.078*	
H24B	0.4397	−0.1236	0.1681	0.078*	
H24C	0.4825	−0.0462	0.1978	0.078*	
O1	1.16091 (15)	0.09328 (9)	0.50350 (16)	0.0475 (4)	
H1	1.1050	0.0897	0.5264	0.057*	
O2	0.82574 (15)	0.18263 (9)	0.11431 (16)	0.0440 (4)	
O3	0.65632 (14)	0.15767 (9)	0.19490 (15)	0.0423 (4)	
H3	0.7021	0.1421	0.1654	0.051*	
O4	0.99402 (16)	0.08135 (9)	0.59430 (16)	0.0464 (4)	
O5	0.59553 (16)	−0.10130 (9)	−0.10871 (17)	0.0499 (5)	
H5A	0.5327	−0.1152	−0.1027	0.060*	
O6	0.54020 (16)	−0.09165 (9)	0.08626 (16)	0.0461 (4)	
Br1	0.80247 (3)	−0.027192 (18)	−0.14649 (3)	0.06469 (13)	

### Atomic displacement parameters (Å^2^)

**Table:**

	*U*^11^	*U*^22^	*U*^33^	*U*^12^	*U*^13^	*U*^23^
C1	0.0487 (16)	0.076 (2)	0.072 (2)	−0.0183 (15)	0.0283 (15)	0.0118 (16)
C2	0.0609 (17)	0.0437 (16)	0.0598 (18)	−0.0099 (14)	0.0214 (14)	−0.0080 (13)
C3	0.0372 (12)	0.0411 (14)	0.0468 (14)	−0.0109 (11)	0.0200 (11)	−0.0024 (11)
C4	0.0404 (13)	0.0394 (14)	0.0424 (13)	−0.0010 (11)	0.0222 (11)	0.0035 (10)
C5	0.0335 (11)	0.0303 (12)	0.0341 (12)	−0.0008 (9)	0.0156 (10)	−0.0040 (9)
C6	0.0285 (10)	0.0265 (12)	0.0351 (12)	−0.0011 (9)	0.0137 (9)	−0.0023 (9)
C7	0.0341 (11)	0.0296 (12)	0.0379 (12)	0.0008 (10)	0.0153 (10)	−0.0018 (10)
C8	0.0280 (11)	0.0456 (15)	0.0456 (14)	−0.0060 (10)	0.0152 (10)	−0.0022 (11)
C9	0.0280 (10)	0.0271 (12)	0.0315 (11)	−0.0016 (9)	0.0105 (9)	0.0011 (9)
C10	0.0311 (11)	0.0259 (12)	0.0352 (12)	−0.0065 (9)	0.0152 (9)	−0.0019 (9)
C11	0.0303 (11)	0.0276 (12)	0.0409 (13)	−0.0081 (9)	0.0141 (10)	−0.0047 (9)
C12	0.0352 (12)	0.0471 (15)	0.0557 (16)	−0.0010 (11)	0.0217 (12)	−0.0104 (12)
C13	0.0421 (13)	0.0542 (17)	0.0528 (15)	−0.0160 (12)	0.0295 (12)	−0.0129 (12)
C14	0.0467 (14)	0.0525 (16)	0.0411 (14)	−0.0143 (12)	0.0255 (12)	−0.0080 (11)
C15	0.0379 (12)	0.0300 (12)	0.0402 (13)	−0.0094 (10)	0.0175 (11)	−0.0027 (10)
C16	0.0684 (19)	0.072 (2)	0.076 (2)	−0.0374 (17)	0.0432 (17)	−0.0181 (17)
C17	0.0594 (18)	0.099 (3)	0.078 (2)	−0.0160 (18)	0.0469 (17)	−0.0260 (19)
C18	0.0276 (10)	0.0262 (12)	0.0342 (11)	0.0003 (9)	0.0107 (9)	−0.0006 (9)
C19	0.0285 (11)	0.0355 (13)	0.0424 (13)	−0.0033 (9)	0.0173 (10)	−0.0054 (10)
C20	0.0345 (12)	0.0436 (14)	0.0375 (12)	0.0020 (10)	0.0188 (10)	−0.0048 (10)
C21	0.0308 (11)	0.0318 (12)	0.0350 (12)	0.0002 (10)	0.0100 (10)	−0.0054 (9)
C22	0.0288 (11)	0.0285 (12)	0.0398 (12)	−0.0036 (9)	0.0125 (10)	0.0015 (10)
C23	0.0344 (12)	0.0310 (12)	0.0331 (12)	−0.0013 (10)	0.0159 (10)	0.0014 (9)
C24	0.0421 (14)	0.0511 (17)	0.0687 (18)	−0.0103 (12)	0.0310 (13)	0.0051 (13)
O1	0.0329 (8)	0.0556 (11)	0.0452 (10)	−0.0014 (8)	0.0115 (8)	0.0117 (8)
O2	0.0321 (8)	0.0451 (10)	0.0447 (9)	0.0018 (7)	0.0101 (7)	0.0096 (8)
O3	0.0345 (8)	0.0473 (10)	0.0418 (10)	0.0010 (7)	0.0153 (7)	0.0044 (8)
O4	0.0424 (9)	0.0513 (11)	0.0361 (9)	0.0028 (8)	0.0110 (8)	0.0070 (8)
O5	0.0421 (9)	0.0560 (11)	0.0485 (10)	−0.0165 (8)	0.0189 (8)	−0.0213 (8)
O6	0.0437 (9)	0.0443 (10)	0.0461 (10)	−0.0162 (8)	0.0179 (8)	−0.0066 (8)
Br1	0.0637 (2)	0.0859 (3)	0.0594 (2)	−0.01735 (16)	0.04152 (16)	−0.02778 (15)

### Geometric parameters (Å, º)

**Table:**

C1—C3	1.530 (3)	C13—C14	1.517 (3)
C1—H1A	0.9600	C13—C16	1.523 (4)
C1—H1B	0.9600	C13—C17	1.528 (4)
C1—H1C	0.9600	C14—C15	1.497 (3)
C2—C3	1.526 (4)	C14—H14A	0.9700
C2—H2A	0.9600	C14—H14B	0.9700
C2—H2B	0.9600	C15—O4	1.286 (3)
C2—H2C	0.9600	C16—H16A	0.9600
C3—C8	1.522 (3)	C16—H16B	0.9600
C3—C4	1.527 (3)	C16—H16C	0.9600
C4—C5	1.496 (3)	C17—H17A	0.9600
C4—H4A	0.9700	C17—H17B	0.9600
C4—H4B	0.9700	C17—H17C	0.9600
C5—O2	1.282 (3)	C18—C19	1.374 (3)
C5—C6	1.402 (3)	C18—C23	1.393 (3)
C6—C7	1.394 (3)	C19—C20	1.384 (3)
C6—C9	1.520 (3)	C19—H19	0.9300
C7—O1	1.284 (3)	C20—C21	1.371 (3)
C7—C8	1.494 (3)	C20—Br1	1.891 (2)
C8—H8A	0.9700	C21—O5	1.355 (3)
C8—H8B	0.9700	C21—C22	1.392 (3)
C9—C10	1.521 (3)	C22—O6	1.369 (3)
C9—C18	1.531 (3)	C22—C23	1.379 (3)
C9—H9	0.9800	C23—H23	0.9300
C10—C11	1.392 (3)	C24—O6	1.390 (3)
C10—C15	1.397 (3)	C24—H24A	0.9600
C11—O3	1.284 (3)	C24—H24B	0.9600
C11—C12	1.494 (3)	C24—H24C	0.9600
C12—C13	1.518 (4)	O1—H1	0.8200
C12—H12A	0.9700	O3—H3	0.8200
C12—H12B	0.9700	O5—H5A	0.8200
			
C3—C1—H1A	109.5	H12A—C12—H12B	107.7
C3—C1—H1B	109.5	C14—C13—C12	107.49 (19)
H1A—C1—H1B	109.5	C14—C13—C16	110.2 (2)
C3—C1—H1C	109.5	C12—C13—C16	111.1 (2)
H1A—C1—H1C	109.5	C14—C13—C17	108.8 (2)
H1B—C1—H1C	109.5	C12—C13—C17	110.0 (2)
C3—C2—H2A	109.5	C16—C13—C17	109.3 (2)
C3—C2—H2B	109.5	C15—C14—C13	114.1 (2)
H2A—C2—H2B	109.5	C15—C14—H14A	108.7
C3—C2—H2C	109.5	C13—C14—H14A	108.7
H2A—C2—H2C	109.5	C15—C14—H14B	108.7
H2B—C2—H2C	109.5	C13—C14—H14B	108.7
C8—C3—C4	107.66 (19)	H14A—C14—H14B	107.6
C8—C3—C2	110.3 (2)	O4—C15—C10	122.4 (2)
C4—C3—C2	110.2 (2)	O4—C15—C14	115.3 (2)
C8—C3—C1	109.7 (2)	C10—C15—C14	122.2 (2)
C4—C3—C1	109.4 (2)	C13—C16—H16A	109.5
C2—C3—C1	109.5 (2)	C13—C16—H16B	109.5
C5—C4—C3	113.19 (19)	H16A—C16—H16B	109.5
C5—C4—H4A	108.9	C13—C16—H16C	109.5
C3—C4—H4A	108.9	H16A—C16—H16C	109.5
C5—C4—H4B	108.9	H16B—C16—H16C	109.5
C3—C4—H4B	108.9	C13—C17—H17A	109.5
H4A—C4—H4B	107.8	C13—C17—H17B	109.5
O2—C5—C6	122.9 (2)	H17A—C17—H17B	109.5
O2—C5—C4	116.1 (2)	C13—C17—H17C	109.5
C6—C5—C4	120.93 (19)	H17A—C17—H17C	109.5
C7—C6—C5	118.1 (2)	H17B—C17—H17C	109.5
C7—C6—C9	118.84 (19)	C19—C18—C23	118.7 (2)
C5—C6—C9	122.94 (18)	C19—C18—C9	121.83 (19)
O1—C7—C6	123.0 (2)	C23—C18—C9	119.05 (19)
O1—C7—C8	114.75 (19)	C18—C19—C20	120.1 (2)
C6—C7—C8	122.2 (2)	C18—C19—H19	120.0
C7—C8—C3	114.66 (18)	C20—C19—H19	120.0
C7—C8—H8A	108.6	C21—C20—C19	122.1 (2)
C3—C8—H8A	108.6	C21—C20—Br1	119.08 (17)
C7—C8—H8B	108.6	C19—C20—Br1	118.78 (17)
C3—C8—H8B	108.6	O5—C21—C20	121.0 (2)
H8A—C8—H8B	107.6	O5—C21—C22	121.3 (2)
C6—C9—C10	113.66 (17)	C20—C21—C22	117.6 (2)
C6—C9—C18	115.23 (17)	O6—C22—C23	125.8 (2)
C10—C9—C18	114.48 (17)	O6—C22—C21	113.30 (19)
C6—C9—H9	103.9	C23—C22—C21	120.9 (2)
C10—C9—H9	103.9	C22—C23—C18	120.5 (2)
C18—C9—H9	103.9	C22—C23—H23	119.7
C11—C10—C15	118.0 (2)	C18—C23—H23	119.7
C11—C10—C9	123.07 (19)	O6—C24—H24A	109.5
C15—C10—C9	118.96 (19)	O6—C24—H24B	109.5
O3—C11—C10	122.9 (2)	H24A—C24—H24B	109.5
O3—C11—C12	115.5 (2)	O6—C24—H24C	109.5
C10—C11—C12	121.6 (2)	H24A—C24—H24C	109.5
C11—C12—C13	113.9 (2)	H24B—C24—H24C	109.5
C11—C12—H12A	108.8	C7—O1—H1	109.5
C13—C12—H12A	108.8	C11—O3—H3	109.5
C11—C12—H12B	108.8	C21—O5—H5A	109.5
C13—C12—H12B	108.8	C22—O6—C24	118.84 (19)
			
C8—C3—C4—C5	51.8 (3)	C11—C12—C13—C16	−69.2 (3)
C2—C3—C4—C5	−68.5 (3)	C11—C12—C13—C17	169.7 (2)
C1—C3—C4—C5	171.0 (2)	C12—C13—C14—C15	−48.5 (3)
C3—C4—C5—O2	145.4 (2)	C16—C13—C14—C15	72.7 (3)
C3—C4—C5—C6	−37.2 (3)	C17—C13—C14—C15	−167.5 (2)
O2—C5—C6—C7	−169.1 (2)	C11—C10—C15—O4	−170.2 (2)
C4—C5—C6—C7	13.7 (3)	C9—C10—C15—O4	8.7 (3)
O2—C5—C6—C9	7.6 (3)	C11—C10—C15—C14	8.6 (3)
C4—C5—C6—C9	−169.5 (2)	C9—C10—C15—C14	−172.5 (2)
C5—C6—C7—O1	172.3 (2)	C13—C14—C15—O4	−160.8 (2)
C9—C6—C7—O1	−4.6 (3)	C13—C14—C15—C10	20.3 (3)
C5—C6—C7—C8	−8.7 (3)	C6—C9—C18—C19	16.9 (3)
C9—C6—C7—C8	174.4 (2)	C10—C9—C18—C19	151.5 (2)
O1—C7—C8—C3	−153.3 (2)	C6—C9—C18—C23	−170.77 (19)
C6—C7—C8—C3	27.6 (3)	C10—C9—C18—C23	−36.2 (3)
C4—C3—C8—C7	−47.4 (3)	C23—C18—C19—C20	1.7 (3)
C2—C3—C8—C7	72.9 (3)	C9—C18—C19—C20	174.1 (2)
C1—C3—C8—C7	−166.4 (2)	C18—C19—C20—C21	−0.4 (3)
C7—C6—C9—C10	93.2 (2)	C18—C19—C20—Br1	177.86 (17)
C5—C6—C9—C10	−83.6 (3)	C19—C20—C21—O5	179.5 (2)
C7—C6—C9—C18	−131.8 (2)	Br1—C20—C21—O5	1.3 (3)
C5—C6—C9—C18	51.4 (3)	C19—C20—C21—C22	−1.3 (3)
C6—C9—C10—C11	87.2 (2)	Br1—C20—C21—C22	−179.51 (17)
C18—C9—C10—C11	−48.1 (3)	O5—C21—C22—O6	0.0 (3)
C6—C9—C10—C15	−91.7 (2)	C20—C21—C22—O6	−179.2 (2)
C18—C9—C10—C15	133.0 (2)	O5—C21—C22—C23	−179.2 (2)
C15—C10—C11—O3	173.1 (2)	C20—C21—C22—C23	1.6 (3)
C9—C10—C11—O3	−5.7 (3)	O6—C22—C23—C18	−179.4 (2)
C15—C10—C11—C12	−5.5 (3)	C21—C22—C23—C18	−0.2 (3)
C9—C10—C11—C12	175.6 (2)	C19—C18—C23—C22	−1.4 (3)
O3—C11—C12—C13	155.0 (2)	C9—C18—C23—C22	−174.0 (2)
C10—C11—C12—C13	−26.3 (3)	C23—C22—O6—C24	−3.6 (3)
C11—C12—C13—C14	51.4 (3)	C21—C22—O6—C24	177.2 (2)

### Hydrogen-bond geometry (Å, º)

**Table:**

*D*—H···*A*	*D*—H	H···*A*	*D*···*A*	*D*—H···*A*
O5—H5*A*···O3^i^	0.82	2.12	2.852 (2)	149
O3—H3···O2	0.82	1.97	2.615 (2)	135
O1—H1···O4	0.82	1.82	2.640 (2)	174

Symmetry code: (i) −*x*+1, −*y*, −*z*.
